# Healthcare use and costs before and after parathyroidectomy in patients on dialysis

**DOI:** 10.1186/1472-6963-13-248

**Published:** 2013-07-02

**Authors:** Vasily Belozeroff, Kerry Cooper, Gregory Hess, Chun-Lan Chang

**Affiliations:** 1 Amgen Inc., One Amgen Center, Dr, Thousand Oaks, CA, 91320, USA; 2 IMS Health, One IMS Drive, Plymouth Meeting, PA, USA

**Keywords:** Secondary hyperparathyroidism (SHPT), Parathyroidectomy, Dialysis, Vitamin D, Hypocalcemia, Healthcare utilization

## Abstract

**Background:**

Parathyroidectomy (PTX) is often performed in dialysis patients when medical treatment fails to control secondary hyperparathyroidism (SHPT). PTX is viewed by many as a cost-containing measure for patients who have been treated with vitamin D analogs and calcimimetics. Yet, information about health resource utilization and costs before and after PTX is limited.

**Methods:**

This retrospective cohort study used professional service and pharmacy claims to identify subjects on dialysis undergoing PTX from 1/1/2008-12/31/2010. Only subjects with at least six months of information before and after PTX were considered. Subjects with primary hyperparathyroidism or kidney transplant were excluded. Prescription use, physician encounters, and surgical complications were compared during the six months immediately before and after PTX.

**Results:**

The mean (SD) age of the 181 study subjects was 51 (15) years; 59% female; and 80% insured by Medicare. Overall, the percentage of patients receiving medications to manage altered mineral metabolism increased from 67% before to 79% after PTX. Specifically, oral vitamin D use increased, while the utilization of cinacalcet decreased resulting in mean (SD) monthly medication charges decreasing from $486 (507) to $226 (288) (*p* < 0.01). The mean (SD) number of physician encounters rose from 15 (14) before to 21 (22) per 6 months after PTX (*p* < 0.01) resulting in the corresponding increase in mean (SD) monthly charges from $1531 (2150) to $1965 (3317) (*p* = 0.08). Hypocalcemia was the predominant diagnosis recorded for post-surgical physician encounters occurring in 31% of all subjects; 84% of hypocalcemic episodes were managed in acute care facilities.

**Conclusions:**

The cost of medications to manage SHPT decreased after PTX largely due to reduction in cinacalcet use, whereas vitamin D use increased likely to manage hypocalcemia. The frequency and cost of physician encounters, especially in acute care settings, were higher in the 6 months after PTX attributable largely to episodes of severe hypocalcemia. Overall, the reduction in prescription costs during the 6 months after PTX is outweighed by the higher costs associated with physician care.

## Background

Secondary hyperparathyroidism (SHPT) associated with alterations in bone and mineral metabolism is common in patients with chronic kidney disease (CKD) [1-3]. SHPT progresses over time manifesting as increasing parathyroid gland hyperplasia and increasing synthesis of parathyroid hormone (PTH). In patients with CKD on dialysis, SHPT is characterized both by elevations in PTH as well as abnormalities in calcium and phosphorus levels. Previous studies [4,5] have reported that these biochemical abnormalities are associated with adverse health outcomes, including elevated rates of bone fracture, cardiovascular disease, and death [6-8]. It is recommended that patients on dialysis should be monitored for serum calcium and phosphorus every 1–3 months and for PTH every 3–6 months [1], while the specific target levels of these biomarkers are still being debated [9-15].

Pharmacological intervention for SHPT includes vitamin D analogs, phosphate binders, and calcimimetics [3,16,17]. Total or partial surgical removal of parathyroid glands, ie, parathyroidectomy (PTX) is considered when patients fail to respond adequately to medical therapy and is recommended in patients with severe HPT defined by PTH > 800 pg/mL [17]. Parathyroidectomy is a viable option for such patients according to the current practice guidelines from the Kidney Disease: Improving Global Outcomes (KDIGO) [18]; it generally improves the short-term and long-term profile of biochemical markers [18-21]. However, the major disadvantage of sub-total PTX are the risks involved in operating in the anterior neck if the patient should need a second surgery; while the disadvantage of total PTX with autotransplant lies in the possibility of prolonged hypocalcemia during the waiting period for the auto transplanted parathyroid tissue to become functional [21]. In a recent, comprehensive review of therapeutic strategies, Stack, BC (2012) noted the decline in parathyroidectomy rates in the US in the 1990s and reported an observed favorable response five years post surgery. Yet, the mortality rate is doubled compared to non-operative SHPT patients and the risk of hypoparathyroidism is also increased, particularly for total PTX with auto-transplantation [19]. Long term consequences of PTX have not been studied sufficiently, mainly due to the ethical issues around potential randomization. However, Kestenbaum et al. (2004) in an observational study of Medicare claims found that patients had higher mortality within 90 days post PTX, but better overall survival 12 months after the surgery compared to those receiving medical therapy to control SHPT [22]. Also, improved survival post-PTX has been reported by Iwamoto et al. [23]. Chen et al. (1998) reported low mortality, short length of hospital stay, and high patient satisfaction among elderly patients undergoing PTX [24].

Based on the literature, PTX appears effective in lowering PTH levels albeit not without risks. However, the current literature remains scarce on the impact of parathyroid surgery on healthcare utilization and costs. The objective of our study was to better understand healthcare utilization and charges before and after PTX among CKD patients on dialysis.

## Methods

This retrospective cohort study was based on the data from private practice provider medical claims (Centers for Medicare and Medicaid Services [CMS]; N = 1500 records), and National Council for Prescription Drug Programs prescription claims (NCPDP v5.2) in the IMS database. The database contains approximately one billion submitted professional services claims per year, and pharmacy claims for dispensed prescriptions from 50%-60% of retail pharmacies across various geographic regions in the United States. All patient-related information is encrypted, de-identified, and compliant with the regulations of the Health Insurance Portability, Affordability and Accountability 1996 (HIPAA 1996).

To be included in the study, patients were required to have at least two professional service claims with ICD-9-CM diagnoses for CKD stage 5 or end stage renal disease (ESRD) at least 30 days apart, and one service claim with the Current Procedural Terminology (CPT) code of 60500, 60502 or 60505 for PTX procedure(s) during January 2008 and December 2010. The index date was the first procedure date of PTX. Further, patients were excluded from the study if they were younger than 18 years of age as of index date, had primary hyperparathyroidism, or kidney transplant, or fewer than six months of observation before and after PTX.

We analyzed, during the six months before and after PTX, the pharmacy claims for drug prescriptions, physician encounters and corresponding charges, and surgical complications recorded in the professional service claims including hematoma or bleeding, vocal cord paralysis or recurrent laryngeal nerve injury, hypocalcemia, wound infection and seroma formation. Descriptive statistics, i.e. means, standard deviation (SD) and median, were reported for continuous variables; the number and percentage of patients were reported for categorical variables. The paired t-tests and McNemar tests were performed to assess the differences before and after PTX. All the statistical analyses were executed with Stata 12.1 (StataCorp LP. College Station, TX).

## Results

The present study identified 181 patients on dialysis undergoing PTX between 2008 and 2010. Their mean (SD) age was 51.1 (14.7) years. Female patients accounted for 59.1% of the study cohort. Almost half of the study cohort inhabited the South census region and 80.1% were insured by Medicare (Table 1).

**Table 1:** Patient demography

	**N**	**%**
**Number of patients**	181	100%
**Age**		
Mean (SD)	51.1	14.7
Median	52	
Age group		
18-44	61	33.7%
45-64	82	45.3%
65-74	31	17.1%
75+	7	3.9%
**Female**	107	59.1%
**Census region**		
Northeast	14	7.7%
Midwest	38	21.0%
South	90	49.7%
West	39	21.5%
**Payer type**		
Commercial	28	15.5%
Medicare	145	80.1%
Medicaid	8	4.4%

Overall the study patients incurred the mean (SD) charges of $4529 (2902) for physician care on the index date – the date receiving PTX. (Table 2) The charges for physician care on index date were mostly billed by surgeon (80.7%) and from inpatient acute care facilities (80.7%).

**Table 2:** Charges of physician care on the index date for PTX

	**Number (%) of patients**		**Charges of physician care on index date**		
	**N**	**%**	**Mean**	**SD**	**Median**
All patients	181	100.0%	4,529	2,902	3,707
Charges of physician care on index date, by physician Specialty					
Surgeon	146	80.7%	3,742	2,060	3,212
Nephrologists	57	31.5%	230	201	158
Primary care	10	5.5%	711	1,595	211
Other specialties	93	51.4%	2,723	2,464	1,901
Charges of physician care on index date, by service place					
Emergent departments/urgent care centers	0	0.0%	NA	NA	NA
Inpatient acute care facilities	146	80.7%	3,702	2,871	3,137
Office/outpatient clinics	3	1.7%	870	849	675
Outpatient hospitals	62	34.3%	3,758	3,006	2,957
Other/unknown service places	15	8.3%	2,914	2,349	2,110

*Abbreviation: NA* not available.

Based on prescription filling activities in the pharmacy claims, more patients received pharmacotherapy to manage SHPT-related biochemical abnormalities after PTX than before (66.9% pre vs 75.8% post; *p* < 0.01) (Table 3). The higher medication use post-surgery was mainly attributed to the increased use of oral vitamin D (calcitriol). The percentage of patients filling scripts for vitamin D increased from 11.6% pre- to 59.1% post-surgery (*p* < 0.01). In contrast, the use of calcimimetics deceased from 34.8% pre- to 11.6% post-surgery (*p* < 0.01). The use of phosphate binders remained stable before and after PTX. While the study patients filled more prescriptions on average post-surgery compared to pre-surgery, their monthly charges in prescriptions filled to manage SHPT decreased from $486 (SD = 507) pre- to $226 (SD = 288) post-surgery (*p* < 0.01).

**Table 3:** Prescription use and charges before and after Parathyroidectomy

	**Before (N = 181)**		**After (N = 181)**		**P value**
**Patients with medication use for: (N,%)**					
Any prescriptions for SHPT management	121	66.9%	142	78.5%	<0.01
Calcimimetics	63	34.8%	21	11.6%	<0.01
Bisphosphonates	0	0.0%	0	0.0%	NA
Phosphate binders	98	54.1%	95	52.5%	0.7
Teriparatide	0	0.0%	0	0.0%	NA
Raloxifen	1	0.6%	0	0.0%	1
Vitamin D	21	11.6%	107	59.1%	<0.01
**Number of filled scripts: (Mean, SD)**					
All prescriptions	22.8	20.4	27.9	23.3	<0.01
Prescriptions for SHPT management	2.6	3.3	3.2	3.2	0.05
**Average monthly charges of scripts filled for: ($; Mean, SD)**					
All prescriptions	445	459	331	358	<0.01
Prescriptions for SHPT management	486	507	226	288	<0.01

Patients had more all-cause physician encounters after PTX than before PTX (Table 4). The mean number (SD) of all-cause physician encounters during a six-month period increased from 15.1 (14.1) pre-surgery to 20.7 (22.0) post-surgery (*p* < 0.01). A higher percentage of patients received acute inpatient care attended by their physician care team post-surgery than pre-surgery (79.0% post vs 43.1% pre; *p* < 0.01); the mean (SD) of six-month physician encounters in the acute inpatient setting increased from 4.5 (9.3) pre-surgery to 10.5 (16.1) post-surgery (*p* < 0.01). However, physician encounters in the acute inpatient setting accounted for a significant increase, whereas fewer patients had encounters in physician offices or outpatient clinics after the surgery (70.2% post vs. 89.0% pre; *p* < 0.01). The average physician encounters in the offices or outpatient clinics is fewer during the six-month period of post-surgery than prior to the surgery (3.5, SD = 4.6 post vs 4.3, SD = 5.1; pre: *p* = 0.03). Healthcare charges billed by physicians also had a marked increase after PTX (Table 5). The mean(SD) monthly charges for all-cause encounters (excluding the actual surgery costs) rose from $1531 (2150) pre-surgery to $1965 (3317) post surgery (*p* = 0.08). The mean (SD) charges in the acute inpatient setting increased significantly from $681 (1474) pre-surgery to $1209 (1706) post-surgery on average (*p* < 0.01). Furthermore, approximately one-third of patients had evidence of possible surgery-related complications identified in the professional service claims. Hypocalcaemia was the most frequently diagnosed condition related to surgery and accounted for 90% of patients with possible surgery-related complications (Table 6).

**Table 4:** Physician care before and after Parathyroidectomy

	**Before (N = 181)**		**After (N = 181)**		**P value**
All-cause encounters in any place					
N (%) of patients w/ ≥1 encounter	181	100.0%	176	97.2%	0.06
Mean (SD) of encounters	15.4	14.1	20.7	22	<0.01
All-cause encounters in emergency departments/urgent care centers					
N (%) of patients w/ ≥1 encounter	37	20.4%	56	30.9%	0.01
Mean (SD) of encounters	0.5	1.4	0.8	1.8	0.05
All-cause encounters in inpatient acute care facilities					
N (%) of patients w/ ≥1 encounter	78	43.1%	143	79.0%	<0.01
Mean (SD) of encounters	4.5	9.3	10.5	16.1	<0.01
All-cause encounters in offices/outpatient clinics					
N (%) of patients w/ ≥1 encounter	161	89.0%	127	70.2%	<0.01
Mean (SD) of encounters	4.3	5.1	3.5	4.6	0.03
All-cause encounters in outpatient hospitals					
N (%) of patients w/ ≥1 encounter	88	48.6%	84	46.4%	0.63
Mean (SD) of encounters	1.5	2.9	1.5	3.1	0.92
All-cause encounters in other/unknown service places					
N (%) of patients w/ ≥1 encounter	105	58.0%	107	59.1%	0.69
Mean (SD) of encounters	4.7	6.5	4.5	6.7	0.7

**Table 5:** Charges related to physician care

	**Before (N = 181)**		**After (N = 181)**		**P value**
**Average monthly charges of encounters for any cause ($):**					
Any service places					
Mean (SD)	1,531	2,150	1,965	3,317	0.08
Median	880		1,093		
Emergent departments/urgent care centers					
Mean (SD)	81	216	155	370	0.01
Median	0		0		
Inpatient acute care facilities					
Mean (SD)	681	1,474	1,209	1,706	<0.01
Median	0		618		
Physician offices/outpatient clinics					
Mean (SD)	920	2,492	1,261	4,680	0.32
Median	250		152		
Outpatient hospitals					
Mean (SD)	587	1,539	603	1,209	0.89
Median	0		0		
Other/unknown service places					
Mean (SD)	440	827	391	531	0.41
Median	273		289		

**Table 6:** Possible complications after surgery

	**N**	**%**
**Number of patients**	181	100%
Patients with any possible surgical complications	61	33.7%
Hematoma/bleeding	5	2.8%
Vocal cord paralysis/recurrent laryngeal nerve injury	1	0.6%
Severe hypocalcaemia	56	30.9%
Wound infection	1	0.6%
Seroma formation	1	0.6%
Mean (SD) of 6-month physician encounters for possible complications	5.5	6.4
Mean (SD) of 6-month total charges of physician encounters for possible complications ($)	1,009	959
Mean (SD) of monthly charges of physician encounters for possible complications ($)	726	654

## Discussions

This study presented the estimates of short-term healthcare utilization and expenses before and after PTX among patients on dialysis in the U.S. The results from our study suggest that after PTX, the overall cost of medications to manage SHPT decreases largely because of the reduction in the use of cinacalcet. However, vitamin D use increases post-surgery, although the net effect on cost is downward because of a shift to greater use of calcitriol as compared to more expensive vitamin D analogues, presumably for the management of hypocalcaemia. The frequency and cost of physician encounters, especially in the acute care setting, were higher in the 6-month period post surgery. Hypocalcaemia was the dominant post-surgical complication, and it was associated with additional physician care, often provided in the acute care setting. Overall, the cost reduction in prescriptions during the 6-month post surgical period was outweighed by the cost increase associated with physician care.

From the clinical standpoint, parathyroidectomy is often reserved for the patients who have failed to respond to medical therapy for SHPT. While clinical practice worldwide is variable with respect to indications for and frequency of utilization of PTX, one recent study reports that the PTX rate in the US has fluctuated between 1992 and 2007 from the lowest recorded in 1998 (7.0 per 1000 patient years) to the highest during 2002 (12.8 per 1000 patient years) [25], which could reflect changing treatment patterns and guidelines.

Parathyroidectomy may also seem appealing from the economic standpoint arguably because of the ability to save downstream costs of pharmacotherapies [26-28]. Our study highlights important short term clinical and economic consequences of PTX which have not been described previously. Although PTX is effective in lowering serum PTH [29-34], it also commonly results in persistent, inappropriately low PTH levels with the inherent risk of hypocalcemia and adynamic bone disease [35-39]. Additionally, about 10-30% of CKD patients undergoing either subtotal or total PTX still experienced persistent or recurrent SHPT after the surgery [22-24,26,27,29].

### Limitations of study

This study has several limitations. Parathyroidectomy is not a very common surgery within the general or CKD population. The study design required at least six months of information before and after PTX; the post PTX cost may have been underestimated. The short study duration was selected because there is no comparator group outside of PTX and the design assumes the intervention of interest. In addition, the study utilized the CPT codes of 60500, 60502 and 60505 to identify patients undergoing PTX. These CPT codes cannot differentiate surgical procedures between total PTX with autotransplantation, and subtotal PTX.

Race, or ethnicity, is generally not available in the professional, medical service claims or pharmacy claims. Thus, it is difficult to evaluate the differences of received treatment and care utilization among different racial or ethnic groups in this study.

This study did not find any claims activities related to the use of IV vitamin D in private practitioner claims or calcium supplement in the pharmacy claims. This is because such patients often receive injectable medications at the dialysis centers making it likely to be recorded in the medical charts in the centers or institutional claims (CMS-1450 form) rather than in professional medical service claims (CMS-1500 form) which was used in this study. Calcium supplements are often available over-the-counter and not included in the prescription formularies.

This study also attempted to bridge the information in the medical and pharmacy claims with laboratory results from a national laboratory provider in order to better understand the changes of clinical endpoints such as serum calcium, phosphorus, PTH and vitamin D before and after PTX. However, there were only few patients who had information available in both claims and laboratory data. It is conceivable that most dialysis patients’ routine blood work is captured within the dialysis centers rather than in facilities outside dialysis centers. Furthermore, the laboratory results are under-reported in the professional service claims (CMS-1500 form) due to the fact most of the laboratory results are not required for reimbursement of professional services.

The professional service and pharmacy claims largely reflect the charges and payments for delivered health services. Neither are designed for research needs to provide patient medical history to assess the disease severity and progression, nor to evaluate cost as these claims tend to be non-adjudicated claims. Finally, the continuum of care may not be fully observed in the claims-based study since treatment may be provided at a healthcare site not reported in the claims or at another practice site.

## Conclusion

Our study showed that cost of medications to manage SHPT decreased after PTX largely due to reduction in cinacalcet use, whereas vitamin D (calcitriol) use increased likely to manage hypocalcemia. The frequency and cost of physician encounters, especially in acute care settings, were higher in the 6 months after PTX attributable largely to episodes of severe hypocalcemia. Overall, the reduction in prescription costs during the 6 months after PTX is outweighed by higher costs associated with physician care. The impact of PTX on postoperative healthcare utilization and charges may be a consideration in the treatment decisions for dialysis patients for whom all medical options have been exhausted.

## Abbreviations

CKD: Chronic kidney disease; CMS: Center for Medicare and Medicaid Services; CPT: Current procedural terminology; ESRD: End-stage renal disease; ICD-9: International classification of diseases, ninth edition, clinical modification; IV: Intravenous; PTH: Parathyroid Hormone; PTX: Parathyroidectomy; SHPT: Secondary hyperparathyroidism; SD: Standard deviation.

## Competing interests

The study was funded by Amgen Inc. VB and KC are employees and shareholders of Amgen. GH and CLC are employees of IMS Health.

## Authors’ contributions

VB and KC conceived the study. All authors contributed to study design. CLC directed the data extraction and management and performed the statistical analyses in consultation with VB, KC and GH. CLC drafted the manuscript and all authors contributed to critically revising the paper. Finally, all authors read and approved the submitted manuscript.

## Pre-publication history

The pre-publication history for this paper can be accessed here:

http://www.biomedcentral.com/1472-6963/13/248/prepub
